# Supplementary material for: Halogenase-Targeted Genome Mining Leads to the Discovery of (±) Pestalachlorides A1a, A2a, and Their Atropisomers
Source: Antibiotics (Basel). 2022 Sep 25;11(10):1304. doi: 10.3390/antibiotics11101304 (PMC9598291; doi:10.3390/antibiotics11101304)
Supplement: Supplementary file 1 [file antibiotics-11-01304-s001.zip › antibiotics-1865450-supplementary.pdf]

Supplementary Material

Halogenase-Targeted Genome Mining Leads to the Discovery of (±) Pestalachlorides A1a, A2a, and Their Atropisomers

Mengna Luo[†], Mengyuan Wang[†], Shanshan Chang, Ning He, Guangzhi Shan* and Yunying Xie*

CAMS Key Laboratory of Synthetic Biology for Drug Innovation, Institute of Medicinal Biotechnology, Chinese Academy of Medical Sciences & Peking Union Medical College, Tiantan xili No.1, Beijing 100050, China

[†] These authors contributed equally to this work

*** Correspondence:**
Yunying Xie
xieyy@imb.pumc.edu.cn (Yunying Xie)
Guangzhi Shan
shanguangzhi@imb.pumc.edu.cn (Guangzhi Shan)

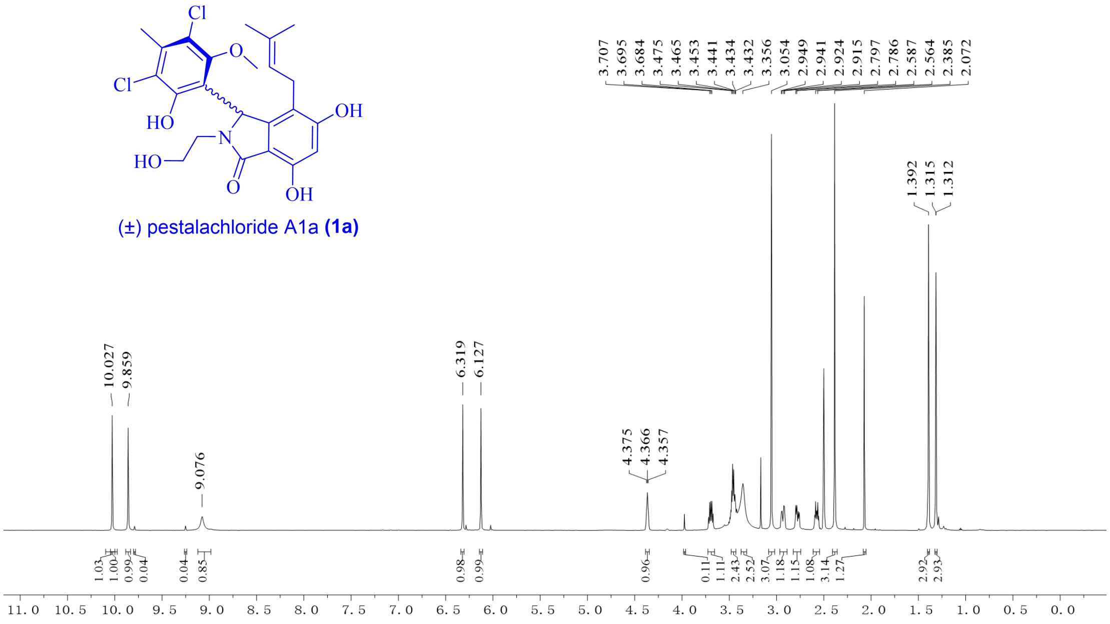

Figure S1. ^{1}H NMR for pestalachloride A1a (**1a**) in DMSO-d_6 (600 MHz)

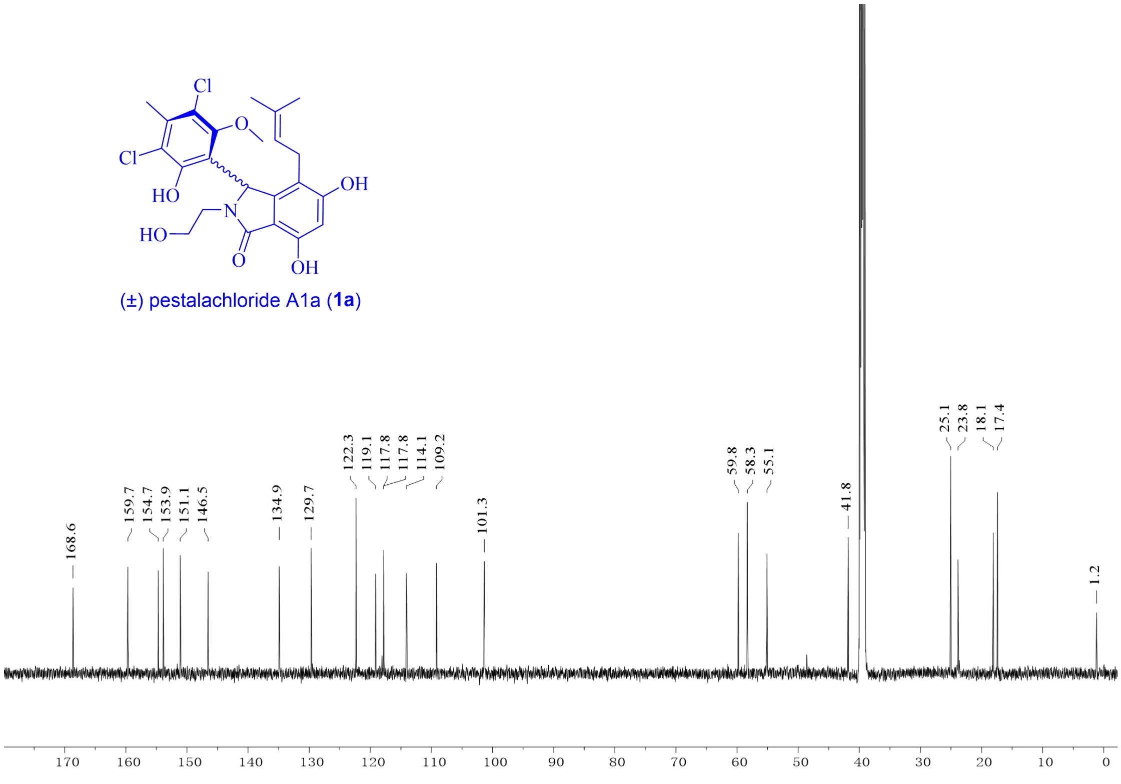

Figure S2. ^{13}C NMR for pestalachloride A1a (**1a**) in DMSO-d_6 (150 MHz)

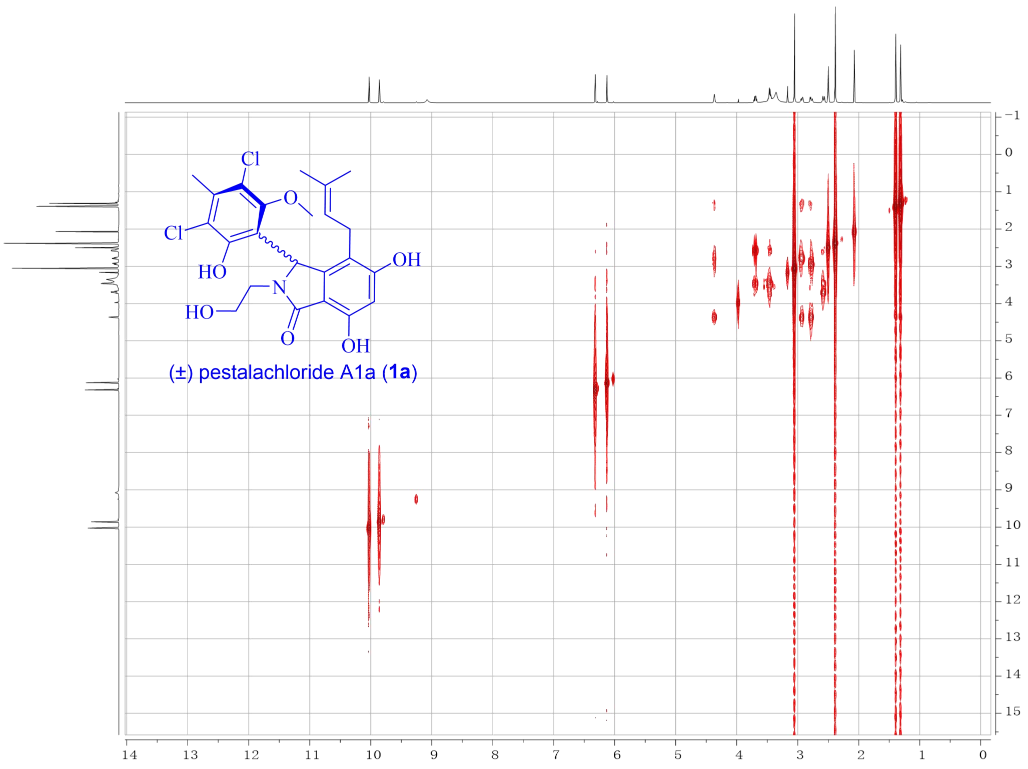

Figure S3. gCOSY for pestalachloride A1a (**1a**) in DMSO-d_6

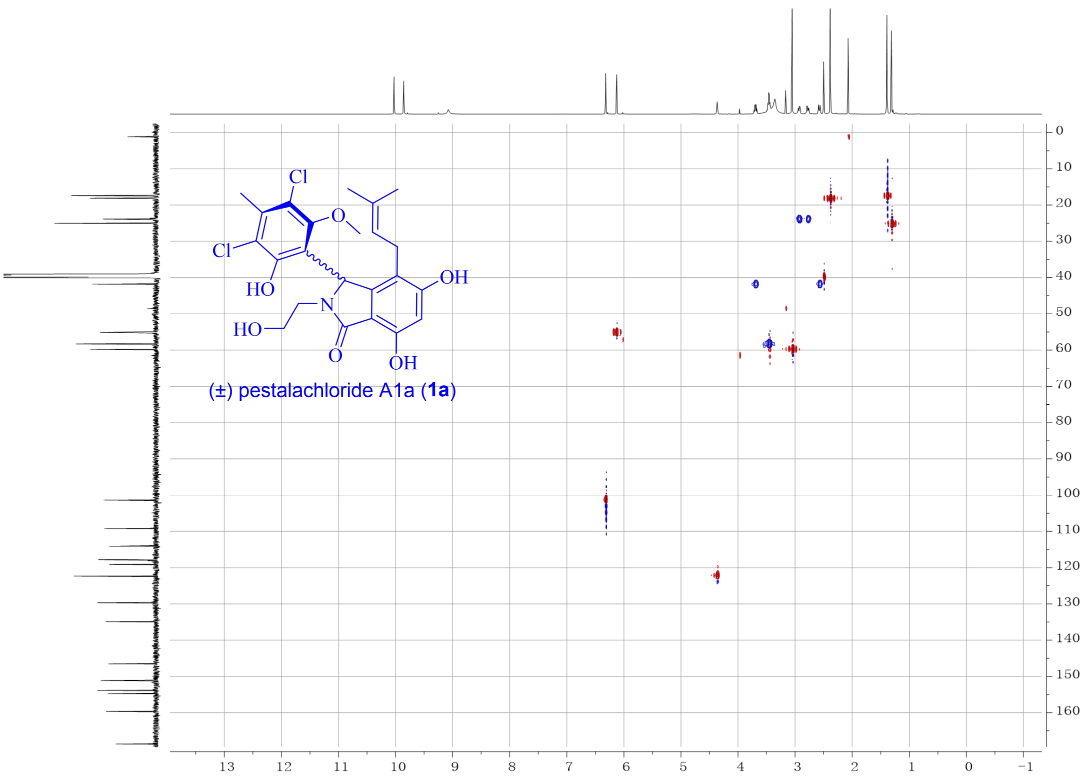

Figure S4. HSQC for pestalachloride A1a (**1a**) in DMSO-d_6

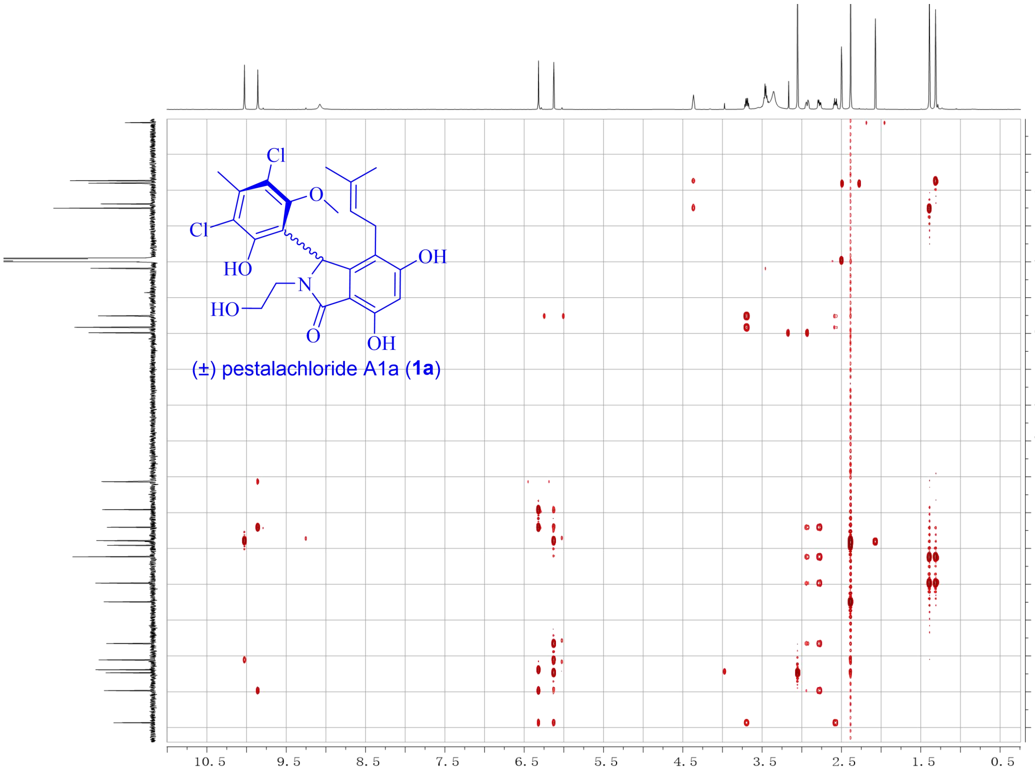

Figure S5. HMBC for pestalachloride A1a (**1a**) in DMSO-d_6

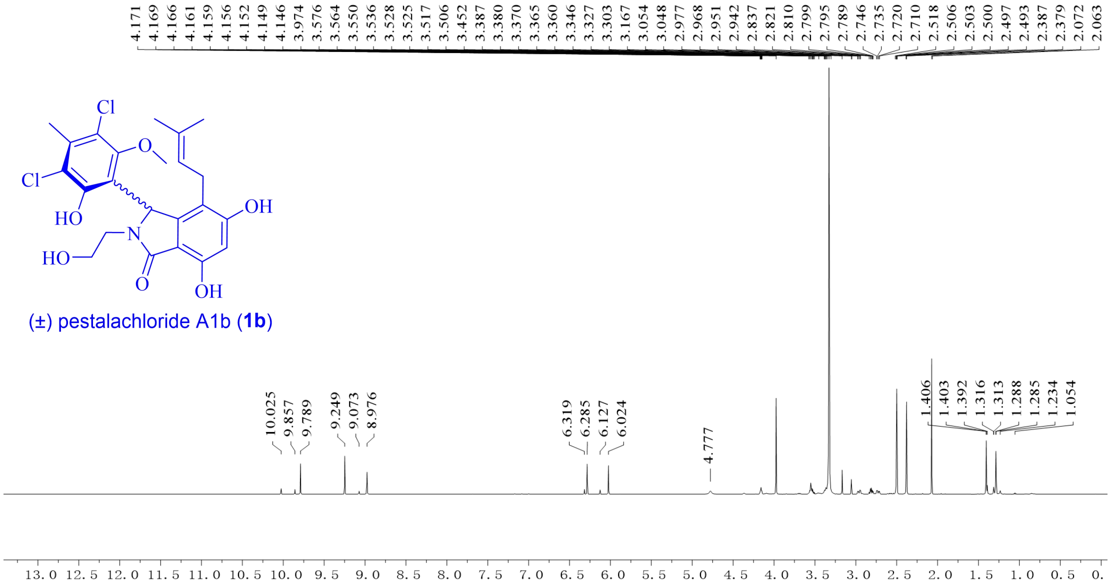

Figure S6. ^{1}H NMR for pestalachloride A1b (**1b**) in DMSO-d_6 (600 MHz)

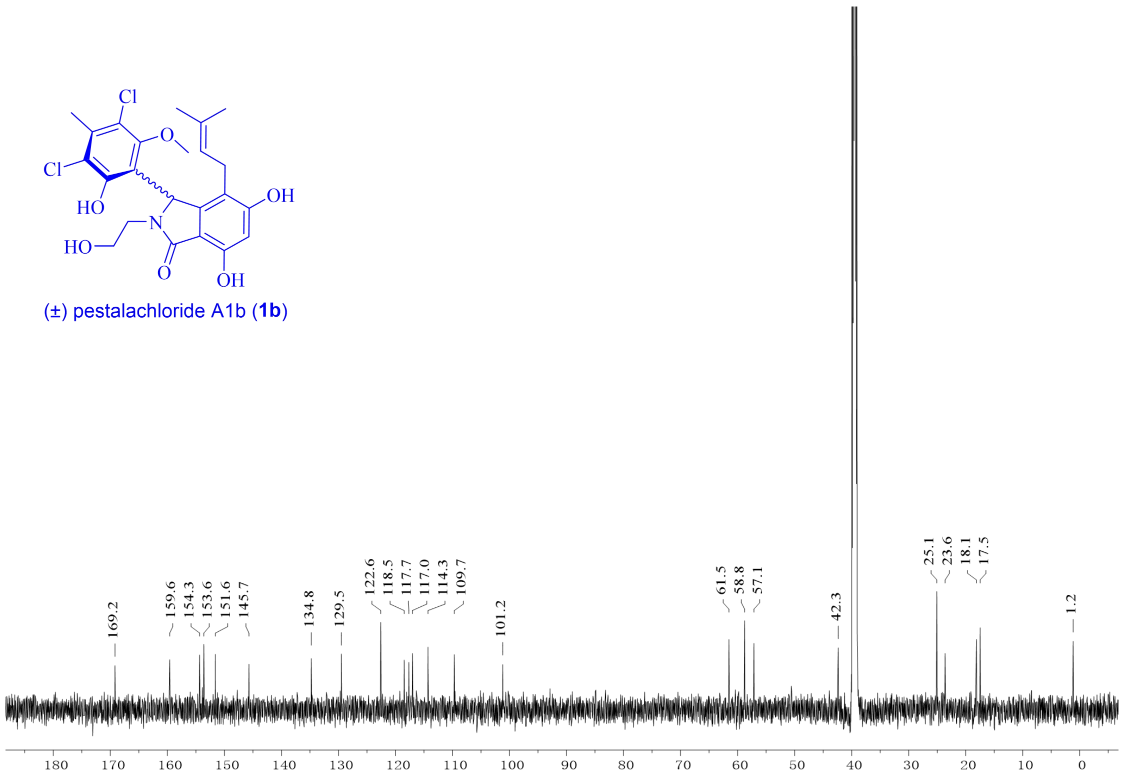

Figure S7. ^{13}C NMR for pestalachloride A1b (**1b**) in DMSO-d_6 (150 MHz)

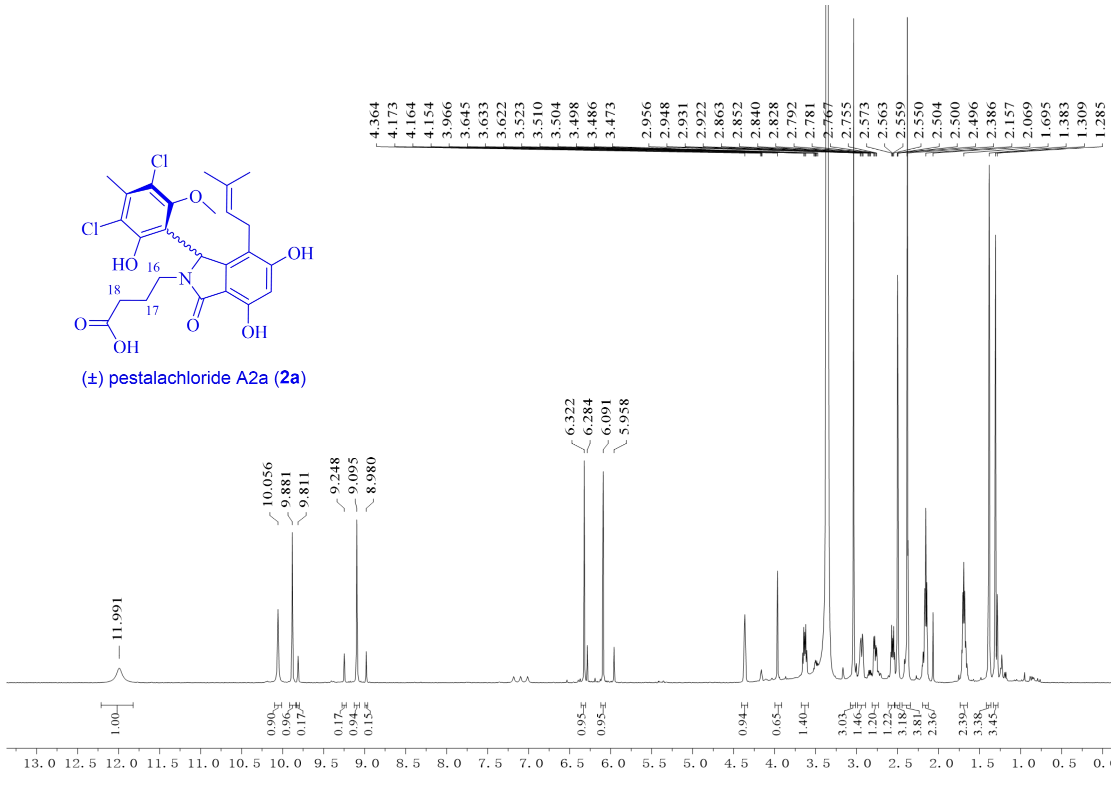

Figure S8. ^{1}H NMR for pestalachloride A2a (**2a**) in DMSO-d_6 (600 MHz)

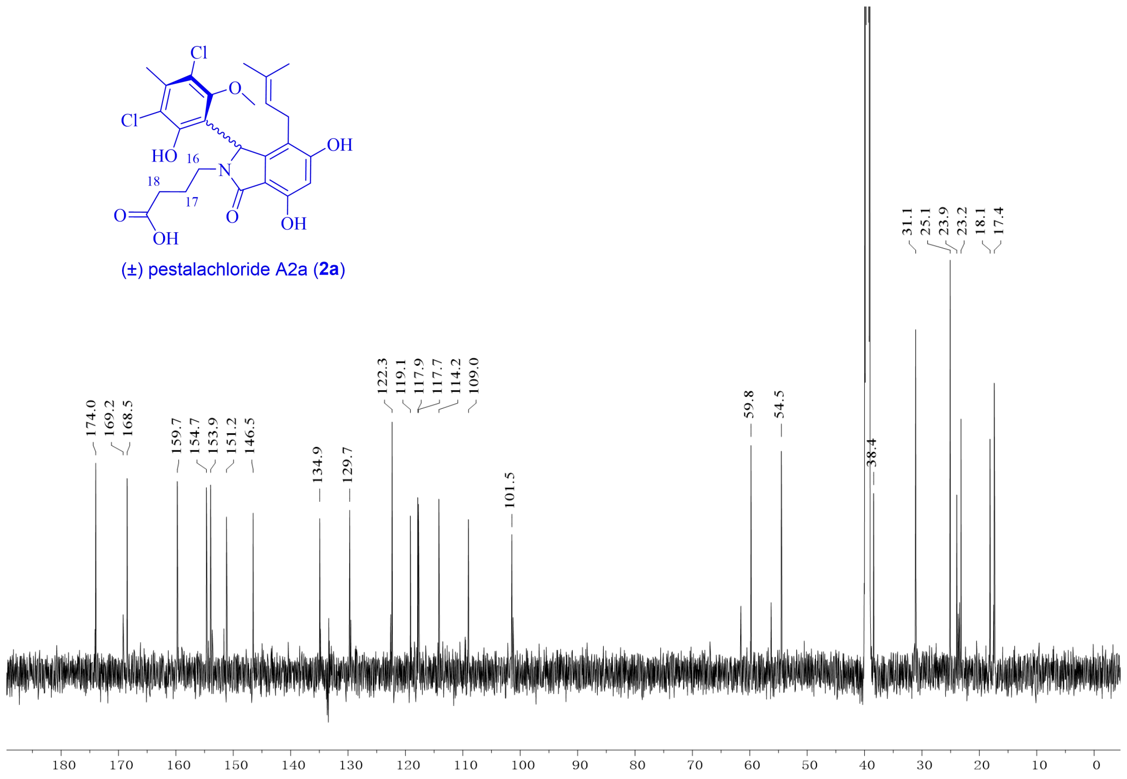

Figure S9. ^{13}C NMR for pestalachloride A2a (**2a**) in DMSO-d_6 (150 MHz)

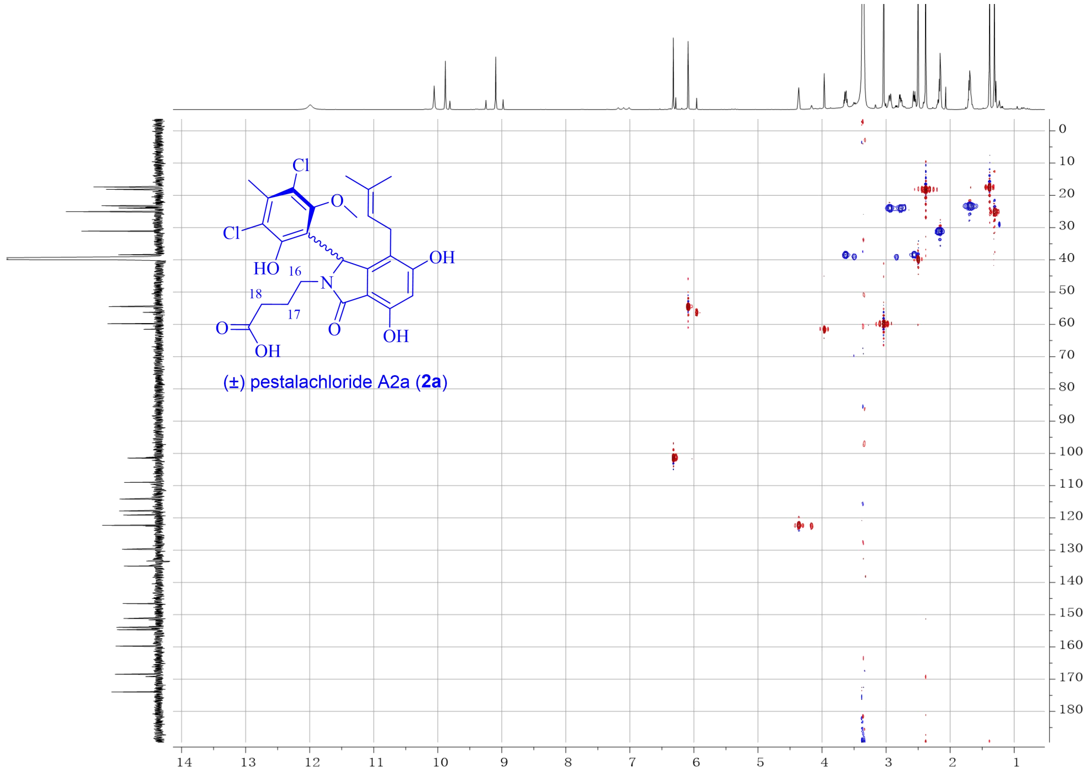

Figure S10. HSQC for pestalachloride A2a (**2a**) in DMSO-d_6

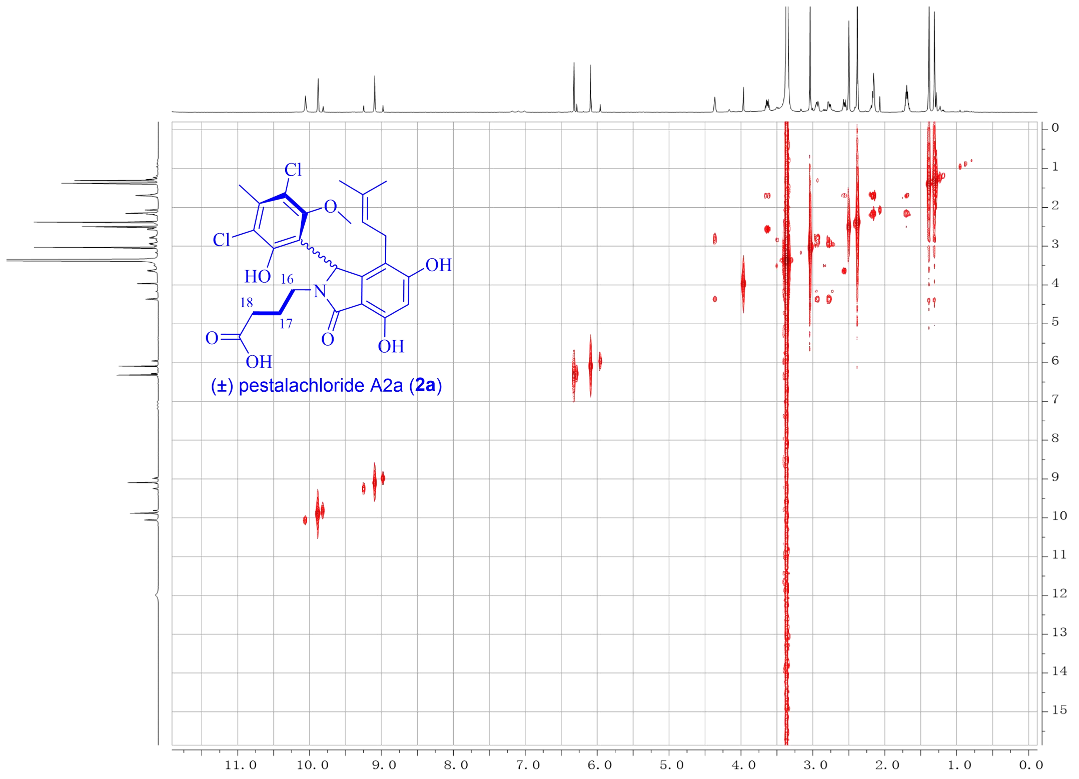

Figure S11. gCOSY for pestalachloride A2a (**2a**) in DMSO-d_6

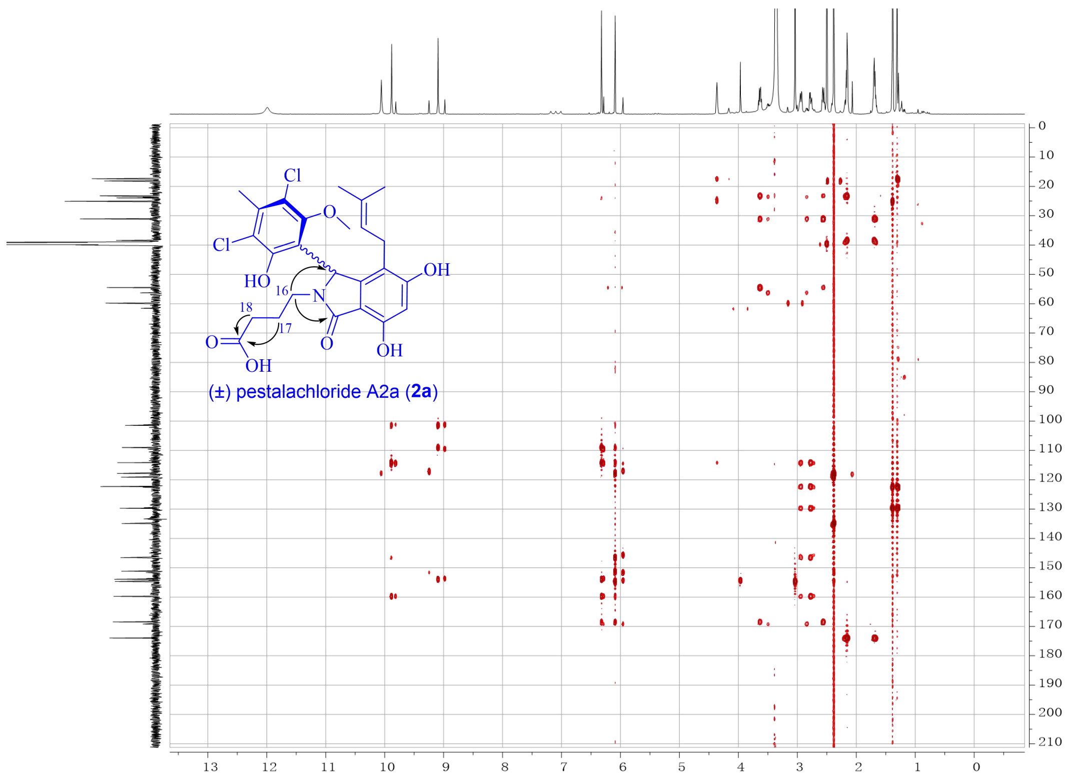

Figure S12. HMBC for pestalachloride A2a (**2a**) in DMSO-d_6

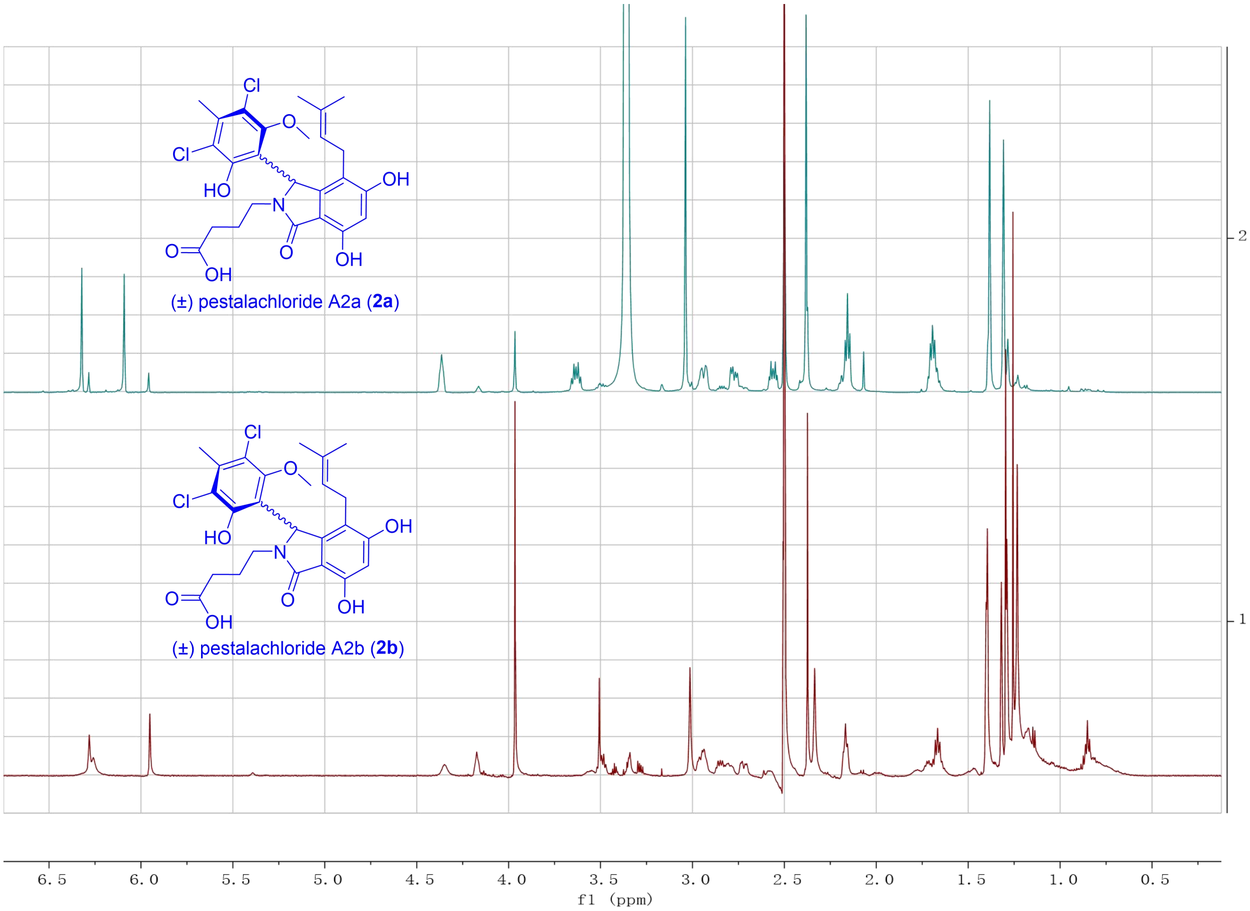

Figure S13. ^{1}H NMR spectrum of pestalachloride A2b (**2b**) by comparison with that of pestalachloride A2a (**2a**) in DMSO-d_6 (600 MHz)

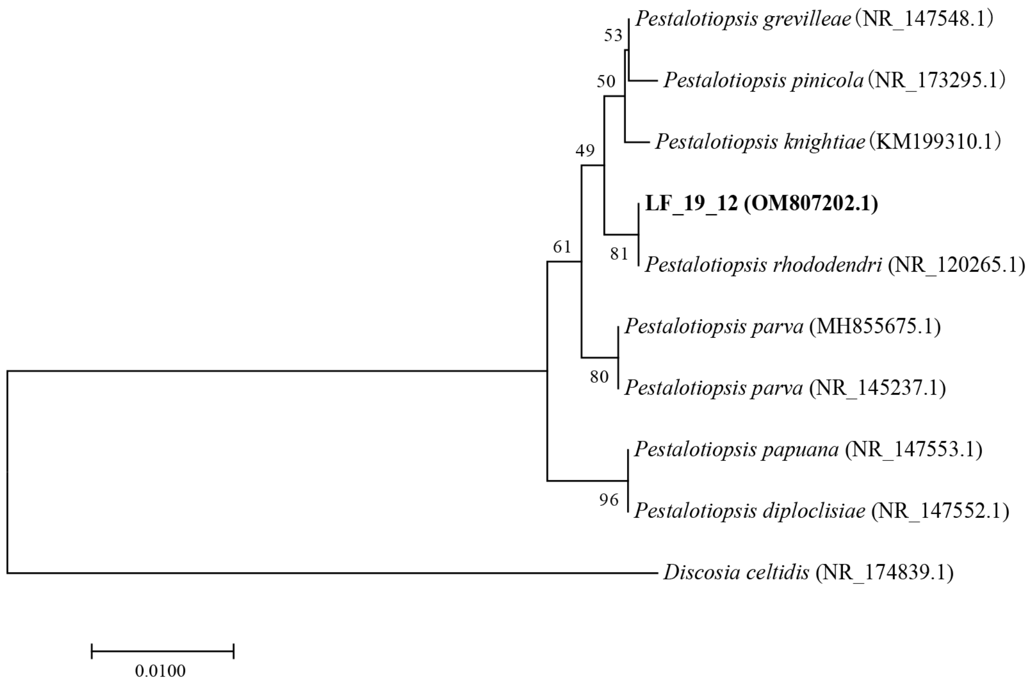

Figure S14. Phylogenetic NJ tree based on ITS squences from LF-19-12 and related type strains . The ITS sequence of *Discosia celtidis* was used as an outgroup. The GenBank accessions are shown in parentheses.
